# Supplementary material for: The muscle-enriched myokine Musclin impairs beige fat thermogenesis and systemic energy homeostasis via Tfr1/PKA signaling in male mice
Source: Nat Commun. 2023 Jul 19;14:4257. doi: 10.1038/s41467-023-39710-z (PMC10356794; doi:10.1038/s41467-023-39710-z)

Supplementary Figures and legends

a

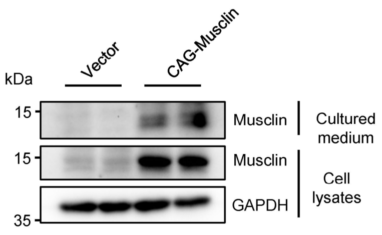

b

Information of patients donating human skeletal muscles

	BMI < 24	24 ≤ BMI < 28	BMI ≥ 28
Age (year)	32.4 ± 13.3	40.0 ± 15.6	35.4 ± 11.3
Sex, *n* (%)			
Woman	3 (30%)	5 (18.5%)	5 (29.4%)
Man	7 (70%)	22 (81.5%)	12 (70.6%)
BW (kg)	63.8 ± 8.0	78.3 ± 10.4	88.5 ± 12.7
Height (cm)	169.7 ± 8.2	174.3 ± 11.8	170.6 ± 9.1
Blood glucose (mmol/L)	4.7 ± 0.6	5.3 ± 0.7*	6.1 ± 2.9

*P = 0.035 (BMI < 24 vs 24 ≤ BMI < 28)

c

Patient information of human skeletal muscles for MUSCLIN protein analysis

	BMI < 24	BMI ≥ 24
Age (year)	32.6 ± 15.49	33.3 ± 14.11
Sex, n (%)		
Woman	2 (40%)	2 (33.3%)
Man	3 (60%)	4 (66.7%)
BW (kg)	60.3 ± 7.36	79 ± 14.07
Height (cm)	166.8 ± 8.64	173.2 ± 16.47
Blood glucose (mmol/L)	5.1 ± 0.50	5.1 ± 0.16

Supplementary Fig. 1 Validation of Musclin secretion and donor information of human skeletal muscles. a Immunoblots of cultured medium and whole cell lysates of HEK293T cells transiently transfected with indicated plasmids. Three independent repetitions were performed with similar results. **b** The basic information of patients donating human skeletal muscle samples analyzed in **Fig. 1e-f**. Two-tailed unpaired Student's *t*-test was used. **c** The information of patient donating human skeletal muscle samples for MUSCLIN protein analysis in **Fig. 1g**.

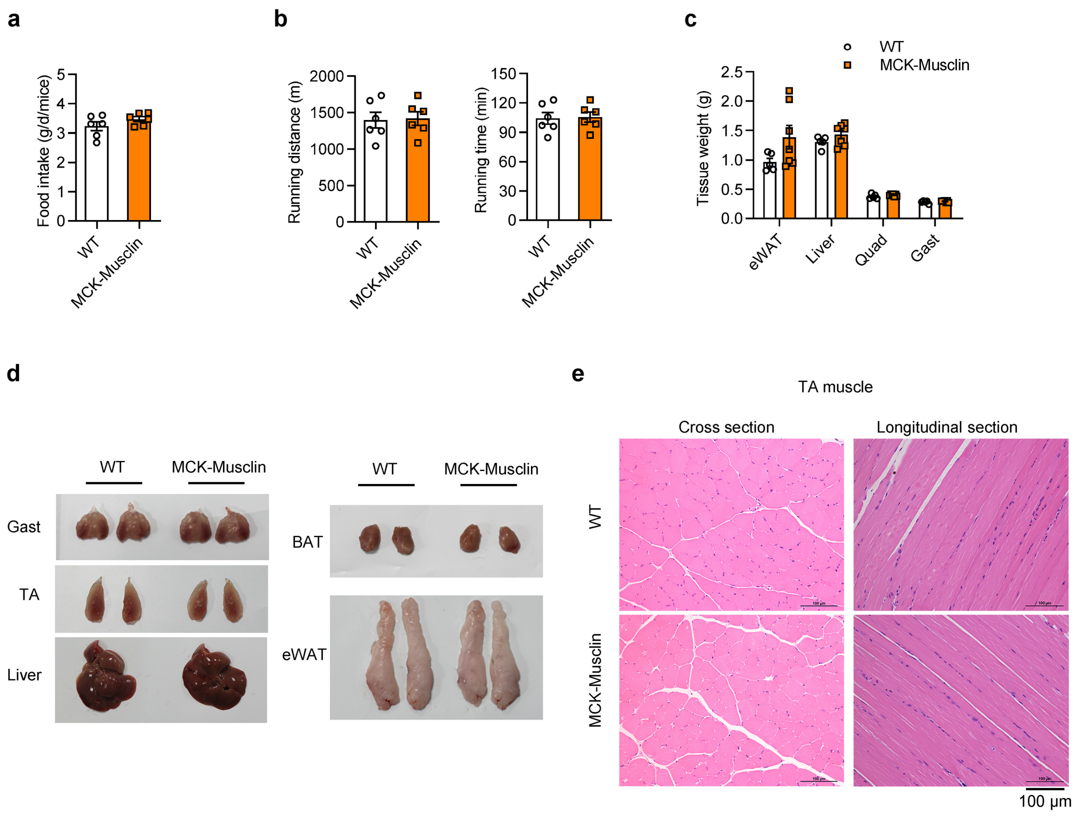

Supplementary Fig. 2 Running performance and muscle morphological studies of WT and MCK-Musclin mice. a-e 6.5 to 7-month-old male mice were used. **a** Food intake of chow diet-fed WT and MCK-Musclin mice. Data represent mean ± SEM (*n* = 6 biologically independent animals per group). **b** Running distance and time of chow diet-fed WT and MCK-Musclin mice before exhaustion. Data represent mean ± SEM (*n* = 6 biologically independent animals per group). **c** Weight of indicated tissues from chow diet-fed MCK-Musclin and WT mice. Data represent mean ± SEM (WT vs MCK-Musclin, *n* = 5 vs 7 biologically independent animals). **d** Representative images of indicated tissues from chow diet-fed WT and MCK-Musclin mice. **e** Representative H&E staining images of TA muscle sections of chow diet-fed WT and MCK-Musclin mice. TA, tibialis anterior. All experiments were performed twice independently with similar results. Source data are provided as a Source Data file.

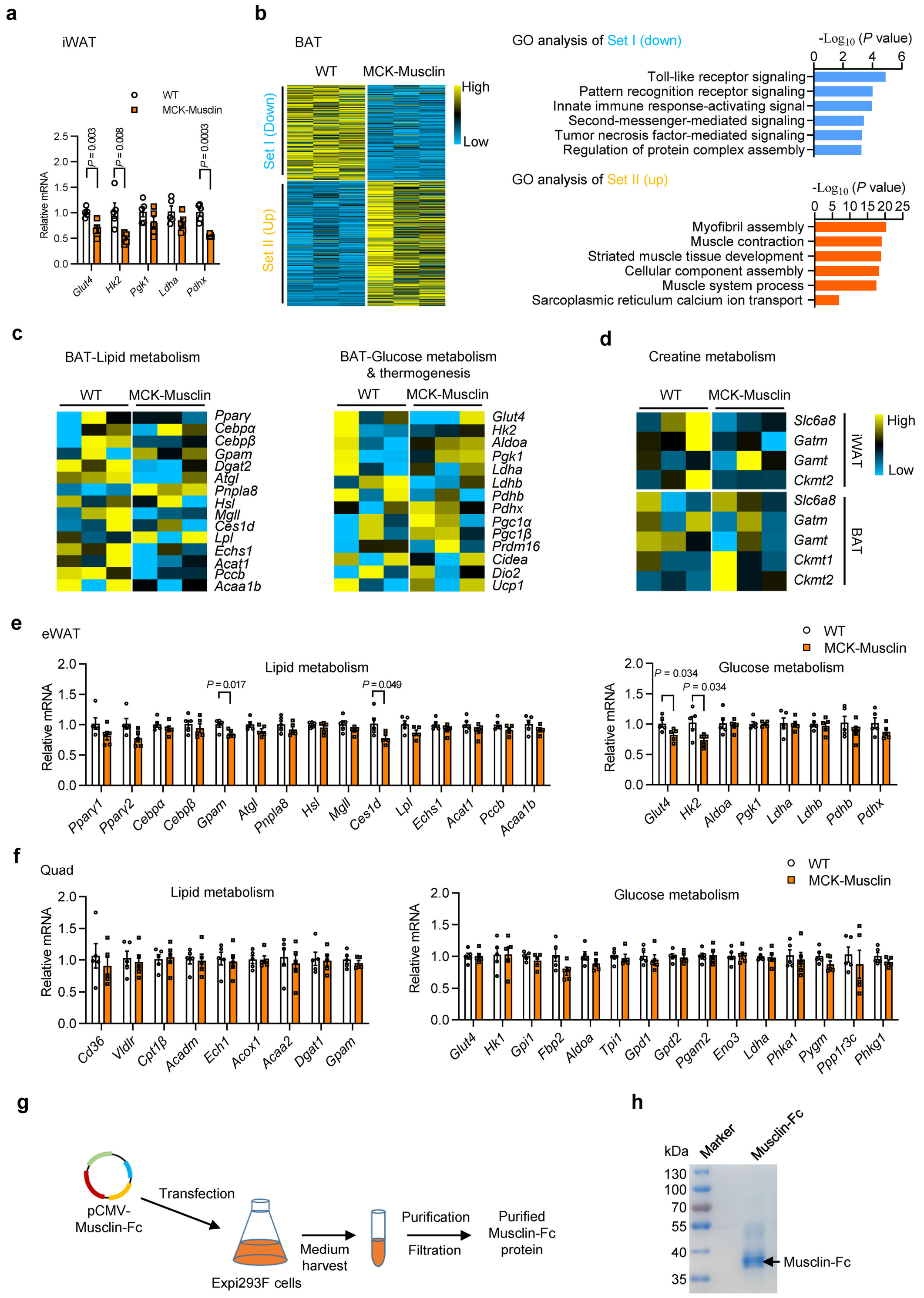

Supplementary Fig. 3 Transcriptional analysis of metabolic tissues in WT and MCK-Musclin mice and purification of Musclin-Fc. Chow diet-fed WT and MCK-Musclin male mice (5 months old) treated with chronic cold exposure at 8°C for 1 week are used in this figure. **a** qPCR

analysis of glucose metabolic gene expression in iWAT. $n = 5$ biologically independent animals per group. **b** Heatmap of differentially expressed genes (DEGs) in BAT RNA-Seq data (Cutoff: $P < 0.05$, $|\log_2(\text{fold change})| > 0.3$) (left), and GO analysis of the genes in the set I (right-top) and set II (right-bottom). **c** Heatmap of lipid & glucose metabolic and thermogenic gene expression in BAT RNA-Seq data. **d** Heatmap of creatine metabolic gene expression in BAT RNA-Seq data. **e** qPCR analysis of gene expression in eWAT. $n = 5$ biologically independent animals per group. **f** qPCR analysis of gene expression in Quad. $n = 5$ biologically independent animals per group. **g** Schematic of workflow on Musclin-Fc protein preparation and purification. **h** Coomassie blue staining of purified Musclin-Fc fusion proteins. Data represent mean ± SEM and two-tailed unpaired Student's t-test was used for data analysis. Differential expression analysis of RNA-Seq data was performed using *Deseq2* package (*P* value by Wald test) in **b-d. a, e, f, h**, experiments were performed for three times independently with similar results. Source data are provided as a Source Data file.

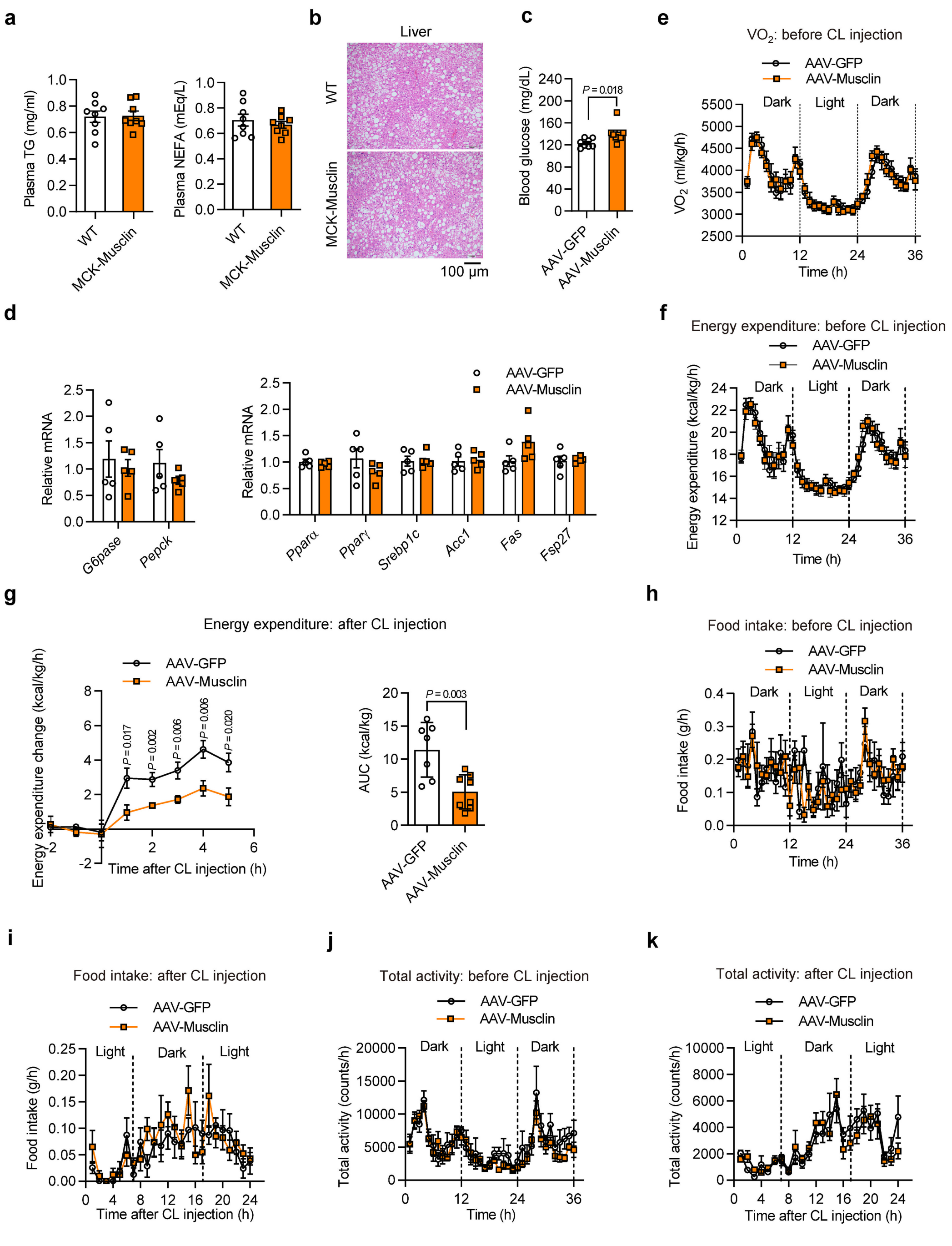

Supplementary Fig. 4 Metabolic studies of Musclin elevated mice. a-b WT and MCK-Musclin male mice were fed with HFD beginning at 3 months old and continued for 3 months. **a** Plasma TG and NEFA. TG, triglycerides; NEFA, nonesterified fatty acids. *n* = 8 biologically independent animals per group. **b** Representative H&E staining image of liver tissues. **c** Blood glucose of chow diet-fed AAV-GFP/Musclin transduced male mice following cold acclimation. *n* = 8 biologically

independent animals per group. AAV-GFP/Musclin were injected at 3.5 months old, and BG was tested after 2 months of AAV injection. **d** qPCR analysis of indicated gene expression in liver from AAV-GFP/Musclin transduced female mice. n = 5 biologically independent animals per group. AAV-GFP/Musclin injection was performed at 4 months old. **e-k** Metabolic cage studies of AAV-GFP/Musclin male mice were fed with HFD for 4 weeks. AAV-GFP vs AAV-Musclin, n = 7 vs 8 biologically independent animals. **e** Oxygen consumption rates before CL injection. **f** Energy expenditure before CL injection. **g** CL-induced increases in energy expenditure, data presented as changes over the values before CL treatment (left) and the AUC (right). **h, i** Food intake before (**h**) and after (**i**) CL injection. **j, k** Total activity before (**j**) and after (**k**) CL injection. Data represent mean ± SEM and the two-tailed unpaired Student's *t*-test was used for statistical analysis. Experiments in **a-d** were repeated independently three times with similar results. Source data are provided as a Source Data file.

a

Patient information of human scWAT

	BMI < 24	BMI ≥ 28	*P* value
Age (year)	63.6 ± 14.6	61.6 ± 11.1	0.7352
Sex, *n* (%)			
Woman	6 (22.2)	2 (28.6)	-
Man	21 (77.8)	5 (71.4)	-
Body weight (kg)	61.0 ± 10.8	84.3 ± 17.3	< 0.001
Height (cm)	164.9 ± 12.0	170.7 ± 8.4	0.2745
Blood glucose (mmol/L)	4.6 ± 0.41	6.7 ± 1.9	< 0.001

b

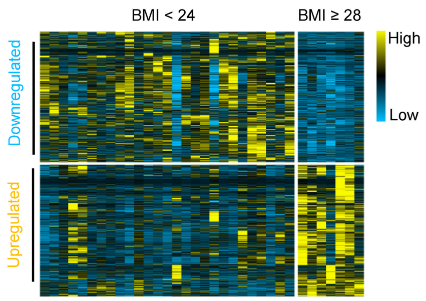

c

GO analysis of human scWAT RNA-Seq data

	Description	$-\mathrm{Log}_{10}$ (*P* value)
Down-regulated	Organic acid catabolism	22.16
	Fatty acid metabolism	15.4
	Cellular amino acid metabolism	13.32
	Lipid oxidation	10.06
	Cellular respiration	6.59
	Temperature homeostasis	4.08
Up-regulated	T cell activation	20.5
	Lymphocyte differentiation	14.17
	Leukocyte cell adhesion	13.82
	Extracellular matrix organization	9.94
	Cytokine production	7.8
	Regulation of cytosolic calcium	7.3

Supplementary Fig. 5 Human scWAT transcriptional profiling analysis. a Patient information of human subcutaneous white adipose tissue (scWAT) samples. Two-tailed unpaired Student's *t*-test was used for statistical analysis. **b** Transcriptomics of scWAT from human subjects with BMI < 24 and BMI ≥ 28. **c** GO analysis of downregulated genes and upregulated genes clustered in (**b**) (Cutoff: $P < 0.05$, $|\log_2 (\text{fold change})| > 0.3$). Differential expression analysis of RNA-Seq data was performed using *Deseq2* package (*P* value by Wald test) in **b.** Source data are provided as a Source Data file.

a

Membrane-associated proteins identified by M.S.

Protein name	Score	MW (kDa)
Transferrin receptor protein 1	37.41	85.7
Caveolae-associated protein 1	28.08	43.9
Neprilysin	16.04	85.6
Cytoskeleton-associated protein 4	9.77	63.7
Extended synaptotagmin-2	9.59	94.1
Catenin beta-1	6.36	85.4
Protein ABHD1	5.1	45.7
Hormone-sensitive lipase	4.34	83.3
Protein ABHD16A	4.16	63
Phosphatidylin ositol 4-kinase type2-alpha	3.88	54.2
P2x purinoceptor 4	2.6	38.1

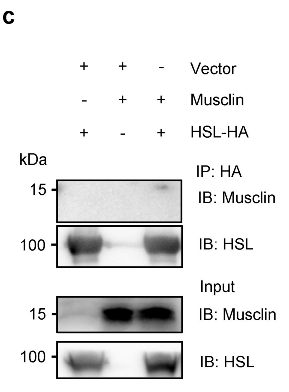

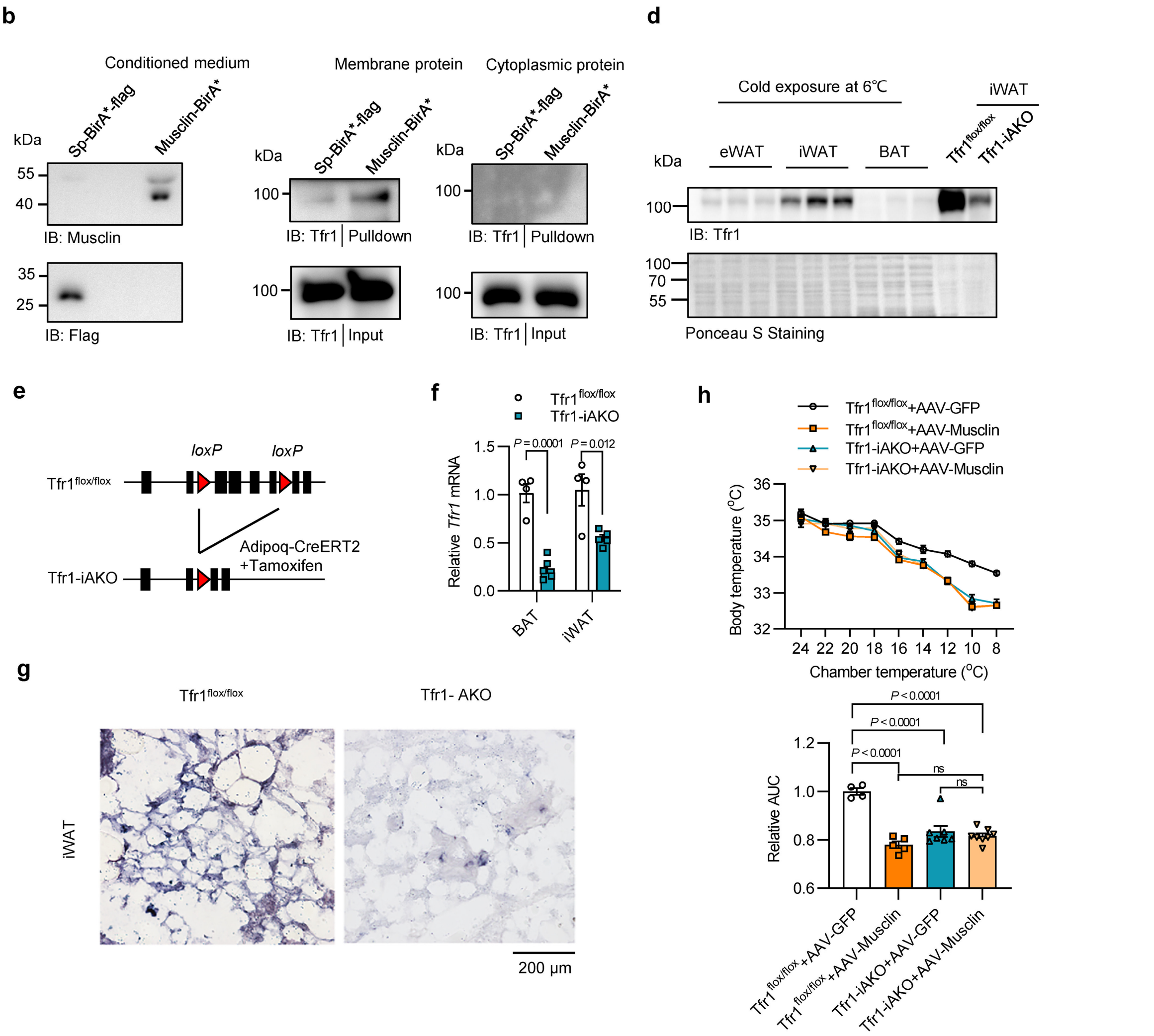

Supplementary Fig. 6 Validation of Tfr1 as the membrane receptor for Musclin in adipocytes. **a** Representative membrane-associated proteins screened from mass spectrometry analysis. **b** Immunoblots of the conditioned medium of HEK293T cells transiently transfected with plasmids

expressing indicated proteins (left), the input and streptavidin-pulldown samples of the cell membrane-associated fractions (middle), and of the cytoplasmic fractions (right). **c** Physical interaction of HSL and Musclin in HEK293T cells transiently transfected with indicated constructs. Musclin, Musclin overexpression plasmids; HSL-HA, HA-tagged hormone-sensitive lipase. **d** Immunoblots of Tfr1 protein in mature adipocytes from eWAT, iWAT, and BAT in male mice after cold exposure at 6°C for 1 week. Total protein lysates of iWAT from TMX-treated Tfr1$^{flox/flox}$ and Tfr1$^{flox/flox}$ Adipoq-CreERT2 (Tfr1-iAKO) mice were used as controls for the validation of the Tfr1 antibody. **e** Schematic representation of the generation of Tfr1$^{flox/flox}$ and Tfr1-iAKO mice. **f** qPCR analysis of *Tfr1* mRNA levels in iWAT and BAT from Tfr1-iAKO and Tfr1$^{flox/flox}$ male mice. Data represent mean ± SEM (Tfr1$^{flox/flox}$ vs Tfr1-iAKO, n = 4 vs 5 biologically independent animals). Two-tailed unpaired Student's t-test was used. **g** Binding signal of SEAP-Musclin fusion protein to iWAT frozen sections from indicated male mice. **h** Core body temperature (top) and relative AUC (bottom) of indicated male mice during cold acclimation. Data represent mean ± SEM (n represents biologically independent animals; 4 for Tfr1$^{flox/flox}$+AAV-GFP, 5 for Tfr1$^{flox/flox}$+AAV-Musclin, 7 for Tfr1-iAKO+AAV-GFP, 8 for Tfr1-iAKO+AAV-Musclin). TMX was injected at 5 months old and AAV-GFP/Musclin was injected at 6 months old, with cold acclimation assay performed 2 weeks after the AAV injection. One-way ANOVA with Tukey's multiple comparisons was used. Tfr1$^{flox/flox}$+AAV-GFP and Tfr1$^{flox/flox}$+AAV-Musclin, Tfr1$^{flox/flox}$ mice treated with TMX followed by AAV-GFP or AAV-Musclin transduction; Tfr1-iAKO+AAV-GFP and Tfr1-iAKO+AAV-Musclin, Tfr1$^{flox/flox}$-Adipoq-CreERT2 mice treated with TMX followed by AAV-GFP or AAV-Musclin transduction. Experiments in **b-h** were repeated independently for three times with similar results. Source data are provided as a Source Data file.

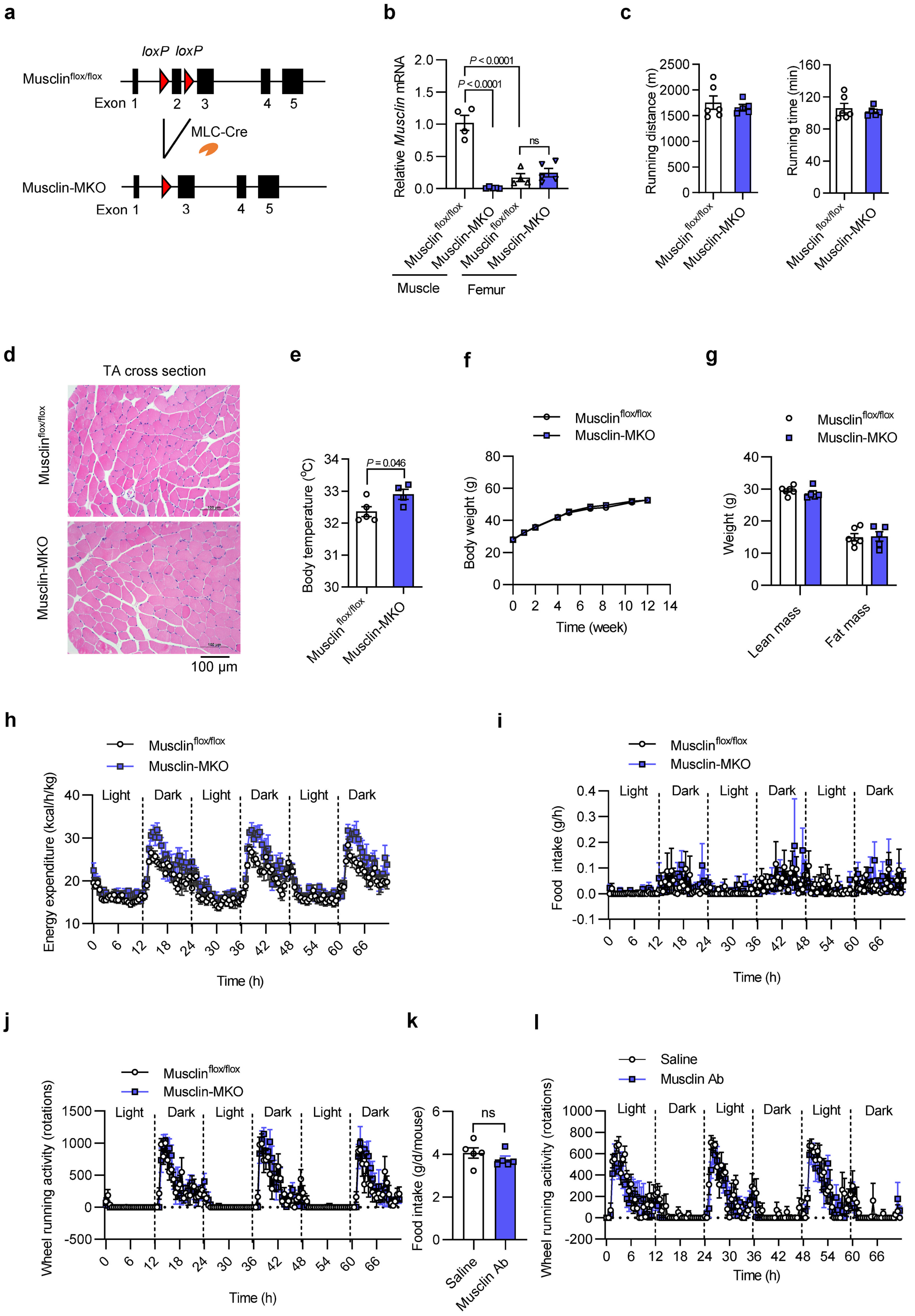

Supplementary Fig. 7 Metabolic analysis in Musclin-inactivated and control male mice. Male mice were used for studies presented in this figure. **a** Schematic of genetic information on Musclin$^{flox/flox}$ and Musclin-MKO mice. **b** qPCR analysis of *Musclin* mRNA levels in skeletal

muscles and femur bone from Musclin$^{flox/flox}$ and Musclin-MKO mice. Musclin$^{flox/flox}$ vs Musclin-MKO, *n* = 4 vs 5 biologically independent animals. **c** Running distance and time of chow diet-fed Musclin$^{flox/flox}$ and Musclin-MKO mice (5 months old) before exhaustion. Musclin$^{flox/flox}$ vs Musclin-MKO, *n* = 6 vs 5 biologically independent animals. **d** Representative H&E staining images of TA muscle sections from Musclin$^{flox/flox}$ and Musclin-MKO mice (*n* = 4 biologically independent animals). **e** Body temperature of chow diet-fed mice (7 months old) following 2 h of acute cold exposure. Musclin$^{flox/flox}$ vs Musclin-MKO, *n* = 5 vs 4 biologically independent animals. **f** Body weight of HFD-fed Musclin-MKO and control mice. Musclin$^{flox/flox}$ vs Musclin-MKO, *n* = 11 vs 9 biologically independent animals. HFD feeding began at 3.5 months old. **g-j** HFD feeding of Musclin$^{flox/flox}$ vs Musclin-MKO mice began at 3 months old. Musclin$^{flox/flox}$ vs Musclin-MKO, *n* = 6 vs 5 biologically independent animals. **g** Body composition analysis of indicated mice following HFD feeding for 3 months. **h** Energy expenditure at room temperature (24°C) for consecutive 3 days following 1 week of thermoneutrality at 30°C. Lean mass was applied for normalization. **i-j** Food intake (**i**) and wheel-running activity (**j**) of mice during metabolic monitoring as in (**h**). **k-l** 2.5-month-old WT male mice were injected with saline or Musclin Ab for 3 months. **k** Food intake. Mice were singly housed and the food intake of each mouse was measured at the same time point each day for 3 days, each symbol represents the average food intake per day for each mouse. *n* = 5 biologically independent animals per group. **l** Wheel-running activity. Saline vs Musclin Ab, *n* = 5 vs 6 biologically independent animals. Data represent mean ± SEM, and one-way ANOVA analysis with Tukey's multiple comparisons (in **b**), two-way ANOVA analysis with Sidak's multiple comparisons (in **f**) two-tailed unpaired Student's *t*-test (in **c, e, g, k**) were used. Experiments in **b-f** were repeated independently three times and experiments in **g-l** were repeated independently twice with similar results. Source data are provided as a Source Data file.

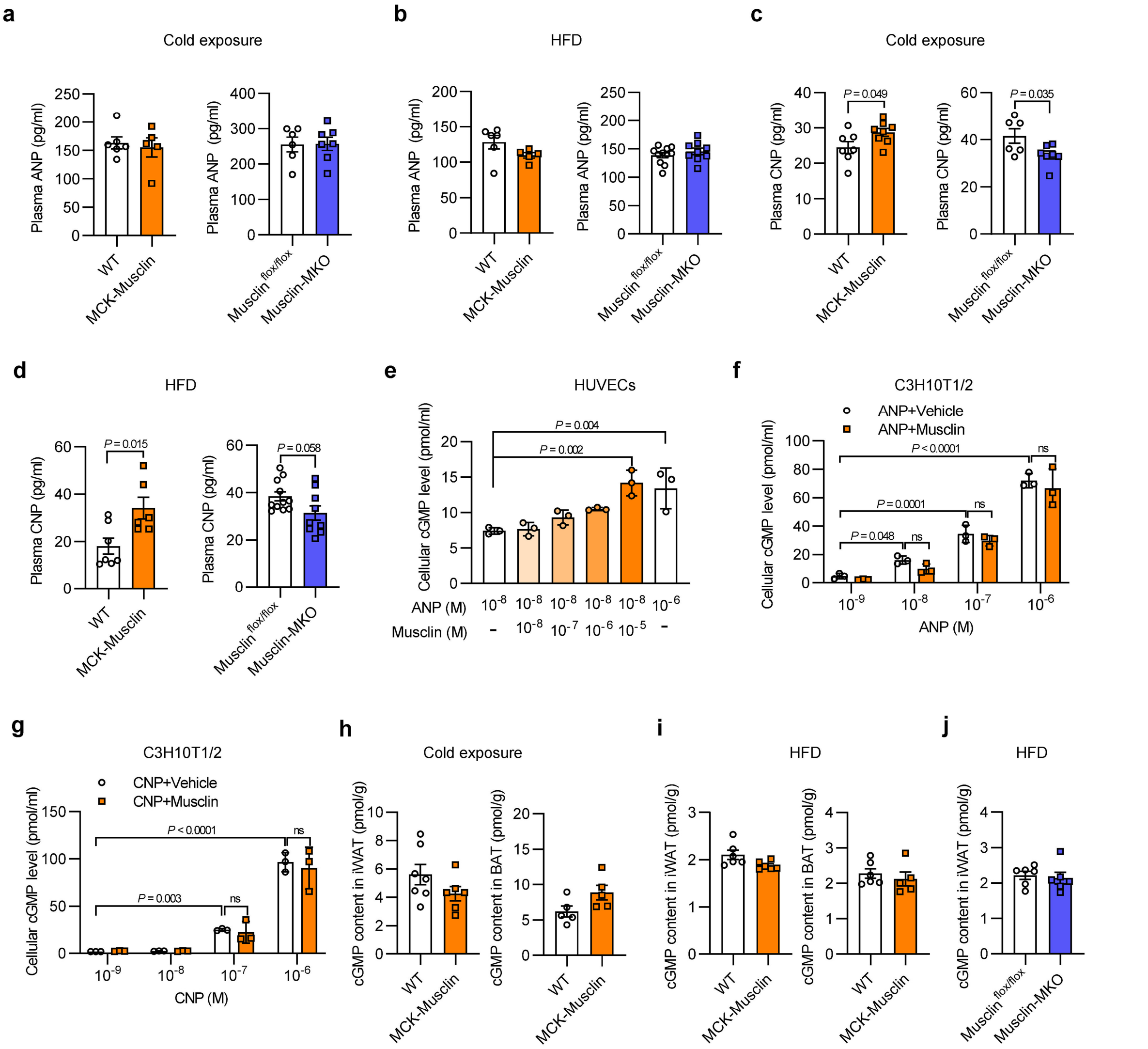

Supplementary Fig. 8 NP/cGMP signaling in thermogenic adipose tissues of male mice and adipocytes upon Musclin manipulation. **a** Plasma ANP levels in chow diet-fed mice. WT (n = 6) and MCK-Musclin (n = 5) mice were chronic cold treated at 8°C for 1 week (left) and Musclin$^{flox/flox}$ (n = 6) and Musclin-MKO (n = 7) were acute cold treated at 4°C (right). **b** Plasma ANP levels in HFD-fed mice. WT vs MCK-Musclin, n = 6 vs 5; Musclin$^{flox/flox}$ vs Musclin-MKO, n = 11 vs 9. **c** Plasma CNP levels in chow diet-fed mice. WT (n = 7) and MCK-Musclin (n = 8) mice were chronic cold treated at 8°C for 1 week (left) and Musclin$^{flox/flox}$ (n = 6) and Musclin-MKO (n = 7) were acute cold treated at 4°C (right). **d** Plasma CNP levels in HFD-fed mice. WT vs MCK-Musclin, n = 7 vs 6; Musclin$^{flox/flox}$ vs Musclin-MKO, n = 11 vs 9. **e** Intracellular cGMP levels in HUVECs after treatment with indicated doses of Musclin-Fc in the presence of ANP (10^{-}

^{8} M). Treatment with 10^{-6} M ANP was used as a positive control (*n* = 3 biologically independent cell samples). **f** Intracellular cGMP levels in differentiated C3H10T1/2-derived adipocytes after treatment with indicated doses of ANP in the presence or absence of Musclin-Fc (0.8 µM) (*n* = 3 biologically independent cell samples). **g** Intracellular cGMP levels in differentiated C3H10T1/2-derived adipocytes after treatment with indicated doses of CNP in the presence or absence of Musclin-Fc (0.8 µM) (*n* = 3 biologically independent cell samples). **h** cGMP content in iWAT (left) and BAT (right) from WT and MCK-Musclin mice after cold treatment at 8°C for 1 week. WT vs MCK-Musclin, *n* = 7 vs 6 (left); *n* = 5 per group (right). **i** cGMP content in iWAT (left) and BAT (right) from HFD-fed WT and MCK-Musclin mice, *n* = 6 per group (left); WT vs MCK-Musclin, *n* = 6 vs 5 (right). **j** cGMP content in iWAT from HFD-fed Musclin$^{flox/flox}$ and Musclin-MKO mice. *n* = 6 per group. **e-g,** data represent mean ± SD; **a-d, h-j,** data represent mean ± SEM (*n* represents biologically independent animals). **e-g,** one-way ANOVA with Tukey's multiple comparisons. **a-d, h-j,** two-tailed unpaired Student's *t*-test. Experiments in **e-g** were repeated independently three times and experiments in **a-d** and **h-j** were repeated independently twice with similar results. Source data are provided as a Source Data file.

Source data for gels presented in Supplementary Figures

Supplementary Fig. 1a

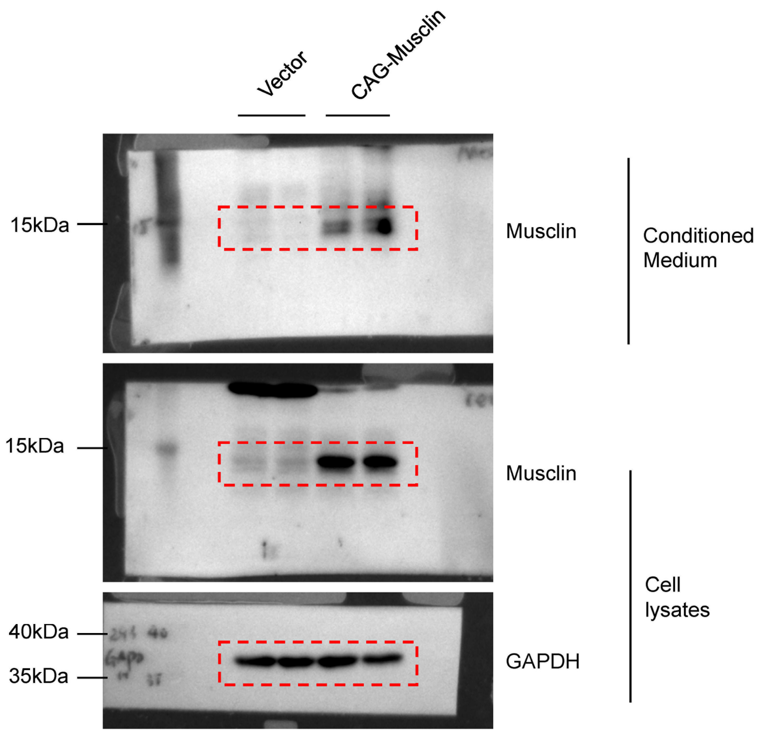

Supplementary Fig. 3h

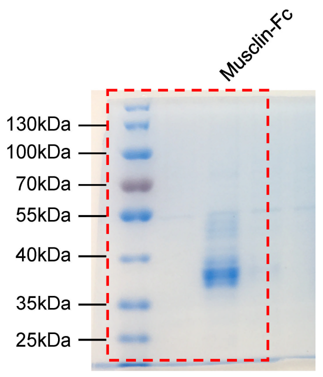

Supplementary Fig. 6b

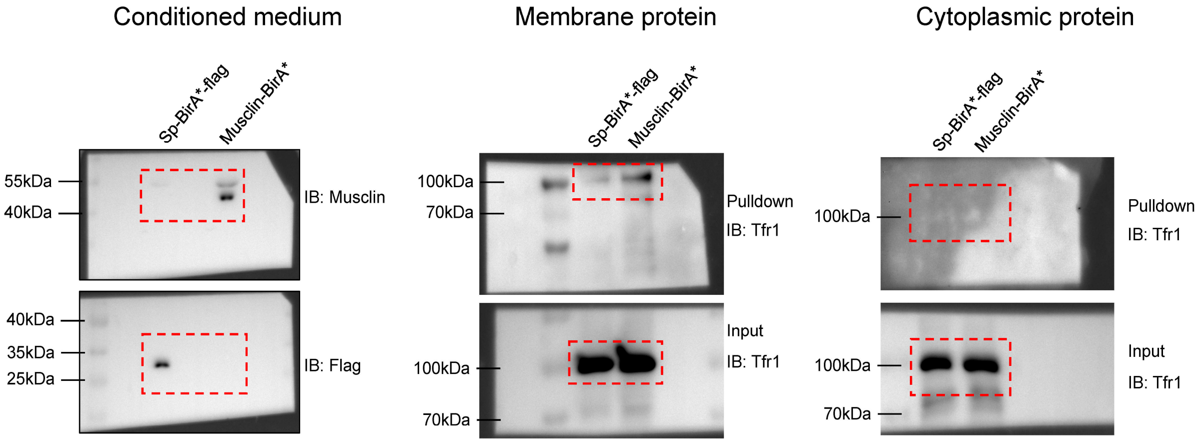

Supplementary Fig. 6c

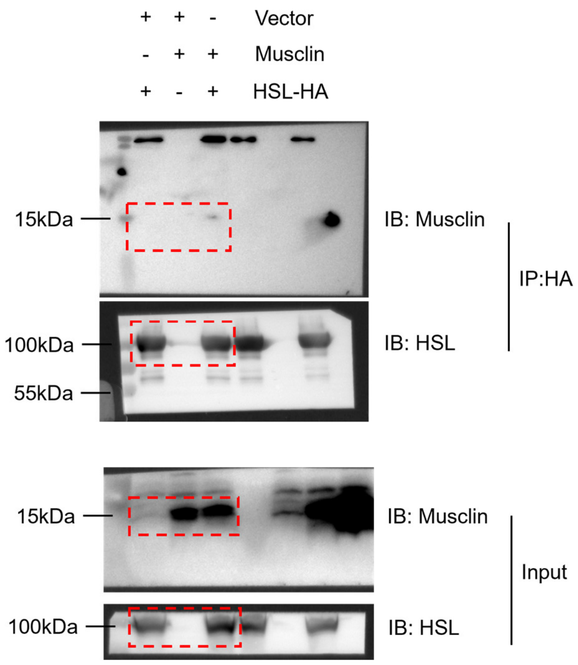

Supplementary Fig. 6d

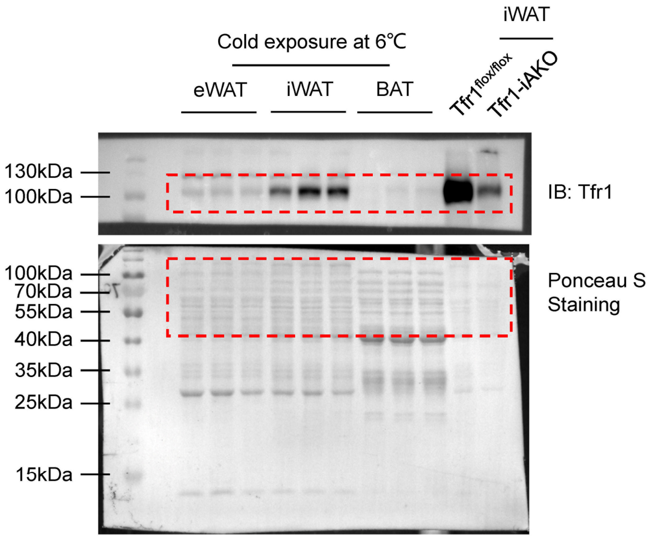

Supplement: Supplementary file 1 — Supplementary Information [file 41467_2023_39710_MOESM1_ESM.pdf]
