# Supplementary material for: The muscle-enriched myokine Musclin impairs beige fat thermogenesis and systemic energy homeostasis via Tfr1/PKA signaling in male mice
Source: Nat Commun. 2023 Jul 19;14:4257. doi: 10.1038/s41467-023-39710-z (PMC10356794; doi:10.1038/s41467-023-39710-z)
Supplement: Supplementary file 3 — Description of Additional Supplementary Files [file 41467_2023_39710_MOESM3_ESM.pdf]

Description of Additional Supplementary Files

Supplementary Data 1: sequences of qPCR primers
