# Supplementary material for: The muscle-enriched myokine Musclin impairs beige fat thermogenesis and systemic energy homeostasis via Tfr1/PKA signaling in male mice
Source: Nat Commun. 2023 Jul 19;14:4257. doi: 10.1038/s41467-023-39710-z (PMC10356794; doi:10.1038/s41467-023-39710-z)
Supplement: Supplementary file 5 — Reporting Summary [file 41467_2023_39710_MOESM5_ESM.pdf]

nature portfolio

Corresponding author(s): Zhuo-Xian Meng

Last updated by author(s): Jun 5, 2023

Reporting Summary

Nature Portfolio wishes to improve the reproducibility of the work that we publish. This form provides structure for consistency and transparency in reporting. For further information on Nature Portfolio policies, see our Editorial Policies and the Editorial Policy Checklist.

Statistics

For all statistical analyses, confirm that the following items are present in the figure legend, table legend, main text, or Methods section.

n/a	Confirmed	
☐	☒	The exact sample size (*n*) for each experimental group/condition, given as a discrete number and unit of measurement
☐	☒	A statement on whether measurements were taken from distinct samples or whether the same sample was measured repeatedly
☐	☒	The statistical test(s) used AND whether they are one- or two-sided<br>*Only common tests should be described solely by name; describe more complex techniques in the Methods section.*
☒	☐	A description of all covariates tested
☐	☒	A description of any assumptions or corrections, such as tests of normality and adjustment for multiple comparisons
☐	☒	A full description of the statistical parameters including central tendency (e.g. means) or other basic estimates (e.g. regression coefficient) AND variation (e.g. standard deviation) or associated estimates of uncertainty (e.g. confidence intervals)
☐	☒	For null hypothesis testing, the test statistic (e.g. *F*, *t*, *r*) with confidence intervals, effect sizes, degrees of freedom and *P* value noted<br>*Give P values as exact values whenever suitable.*
☒	☐	For Bayesian analysis, information on the choice of priors and Markov chain Monte Carlo settings
☒	☐	For hierarchical and complex designs, identification of the appropriate level for tests and full reporting of outcomes
☒	☐	Estimates of effect sizes (e.g. Cohen's *d*, Pearson's *r*), indicating how they were calculated

Our web collection on statistics for biologists contains articles on many of the points above.

Software and code

Policy information about availability of computer code

Data collection	NovaSeq 6000 (Illumina) for RNA sequencing.<br>LightCycler480 (Roche) for quantitative Real-time PCR.<br>MiniChemi™610 (Mini Chemiluminescent/Fluorescent Imaging and Analysis System) with Sage Capture™ Imaging Software was used for immunoblot data collection.<br>NIS Elements F 4.60 (Nikon) for H&E image collection.<br>CellSens Standard (Olympus) for SEAP binding assay data collection.<br>Seahorse Wave Desktop and Controller 2.6.1 (Agilent) for oxygen consumption rate (OCR) from adipocytes.<br>Comprehensive Lab Animal Monitoring System (CLAMS, Columbus Instruments) for collection of energy expenditure data from AAV-GFP/Musclin mice; PhenoMaster (TSE systems) for collection of energy expenditure data from Musclin-MKO & CTR and Musclin Ab/saline-treated mice.<br>Proteome Discoverer version 1.4 (Thermo Scientific™) for mass spectrometry data analysis.<br>Seahorse Wave Desktop and Controller 2.6.1 (Agilent) was used for data acquisition of OCR assay.
Data analysis	GraphPad Prism 9 was used to generate graphs and perform statistical analysis in this study.<br><br>For 7 tissues from C57BL/6J WT mice, iWAT and BAT from MCK-Musclin and control mice , iWAT from cold-acclimated mice and room temperature-housed control mice (wildtype) and human muscles, raw reads of RNA sequencing were filtered with fastp (V0.20.0, https://github.com/OpenGene/fastp) and bowtie2 (V2.3.5, https://bowtie-bio.sourceforge.net/bowtie2/index.shtml). Filtered data were then aligned with HISAT2 (V2.1.0, https://daehwankimlab.github.io/hisat2/download/) to mm10 reference genome (Mus_musculus.GRCm38.90, https://hgdownload.soe.ucsc.edu /goldenPath/mm10/bigZips/) (for mouse tissue data) or to Homo_sapiens.GRCh38.p13 (for human data). The FPKM (fragments per kilobase of exon per million fragments mapped) or TPM (transcripts per million reads) values were calculated using StringTie

(V2.0, https://ccb.jhu.edu/software/stringtie/) to determine gene expression levels.

For human scWAT, the sequencing data was filtered with SOAPnuke (V1.5.6) to obtain clean data which were then aligned to reference genome (GCF_000001405.38_GRCh38.p12) with HISAT2 (V2.1.0). Expression level of gene (FPKM value) was calculated by RSEM (V1.3.1, http://deweylab.biostat.wisc.edu/rsem) to determine gene expression levels.

For all these sequencing data, differential expression analysis was performed using the Deseq2 (v.1.20.0) package (P value by Wald test). Gene Ontology (GO) and pathway grouping and enrichment studies were performed by clusterProfiler (V3.12.0, https://bioconductor.org/packages/release/bioc/html/ clusterProfiler.Html). Results were visualized by ggplot2 (V3.2.1, https://tidyverse.github.io/ggplot2-docs/index.html) and pheatmap (V1.0.10, https://cran.r-project.org/web/packages/pheatmap/index.html).

Image J (National Institute of Health) version 1.52q was used for quantification analysis of western blot and H&E images.
FLIR Tools 5.7 was used for the quantification of average body surface temperature.
All packages and codes used in this study are open-source and publicly available.

For manuscripts utilizing custom algorithms or software that are central to the research but not yet described in published literature, software must be made available to editors and reviewers. We strongly encourage code deposition in a community repository (e.g. GitHub). See the Nature Portfolio guidelines for submitting code & software for further information.

Data

Policy information about availability of data

All manuscripts must include a data availability statement. This statement should provide the following information, where applicable:

- Accession codes, unique identifiers, or web links for publicly available datasets
- A description of any restrictions on data availability
- For clinical datasets or third party data, please ensure that the statement adheres to our policy

RNA-Seq data of 7 tissues from C57BL/6J WT mice, and the RNA-Seq data of iWAT and BAT from MCK-Musclin and control mice are available in GEO database under accession code: GSE228218 (https://www.ncbi.nlm.nih.gov/geo/query/acc.cgi?acc=GSE228218); RNA-Seq data of iWAT from cold-acclimated mice and control mice are available in SRA database under accession code: PRJNA948712 (https://www.ncbi.nlm.nih.gov/bioproject/PRJNA948712). RNA-Seq datasets of human muscle samples and of human scWAT samples have been deposited in the Genome Sequence Archive in National Genomics Data Center, China National Center for Bioinformation / Beijing Institute of Genomics, Chinese Academy of Sciences (GSA-Human: HRA004352) that are publicly accessible at https://ngdc.cncb.ac.cn/gsa-human/browse/HRA004352 (the MOST Approval number: 2023BAT0826). The mass spectrometry proteomics data have been deposited to the ProteomeXchange Consortium via the PRIDE partner repository with the dataset identifier PXD041019 (https://www.ebi.ac.uk/pride/archive/projects/PXD041019), which can be freely accessed.

The images representing human participants, mouse models, skeletal muscle, myotube, adipose tissue, RNA, sequencing, qpcr analysis, culture dish and adipocyte shown in Fig. 1a, 3a, 5a, 6d, 8a and Supplementary Fig. 1b, 5a are created with BioRender.com.

All other data and image files are available from the “Source Data” file. Source data are provided with this paper.

Human research participants

Policy information about studies involving human research participants and Sex and Gender in Research.

Reporting on sex and gender	Tissue and plasma samples from both male and female donors were used in this study. Overall, 54 (41 male and 13 female) human skeletal muscle samples, 34 (26 male and 8 female) human scWAT samples, and 144 (89 male and 55 female) human plasma samples were included. According to the BMI criteria by the Working Group on Obesity in China (WGOC), human samples were divided into control (BMI < 24), overweight (24 ≤ BMI < 28), and obesity (BMI ≥ 28) groups. Sex was comparable between groups, as shown in Supplementary Figure 1b-c and Supplementary Figure 5a. Sex information for the human samples shown in the main Figures 1b, 1e-f, 1g, 1h are provided in Source Data.
Population characteristics	Human biological samples including skeletal muscle (from a total of 54 donors; data shown in Figure 1b, 1e-f, 1g, and Supplementary Figure 1b-c), scWAT (from a total of 34 donors; data shown in Supplementary Figure 5a-c), and plasma (from 144 donors, data shown in Fig. 1h) were analyzed in this study. According to the BMI criteria by the Working Group on Obesity in China (WGOC), human samples were divided into control (BMI < 24), overweight (24 ≤ BMI < 28), and obesity (BMI ≥ 28) groups. Sex and age were comparable between groups. Clinical characteristics of population donating skeletal muscles and scWAT are summarized in Supplementary Figure 1b-c and Supplementary Figure 5a.
Recruitment	The human muscle samples analyzed were gracilis and semitendinosus muscles collected between 2018-2021 from patients undergoing ligament repair or reconstruction treatment due to ligamentous injury of the knee joint or patella injury at the First People's Hospital of Lianyungang City. Donors with BMI < 18, deficiency in muscle development, skeletal muscle-related diseases or displaying disability during normal physical activity, or those with abnormal body height were all excluded from this study. Subjects with other severe diseases, including malignant tumors and severe obesity-independent cardiovascular diseases, were also excluded. Moreover, to minimize the confounding effect of previous injury in the current findings, the human muscle samples were only collected from patients who have fully recovered from acute injury with confirmation of the absence of acute inflammation prior to ligament repair or reconstruction treatments. BMI is the only criterion to divide the human muscle samples into different groups, as such, we assumed that the physical activity should be similar between groups. The scWAT samples were collected between 2019-2020 from the fat layer that is located underneath the Scarpa's Fascia and outside of the abdominal cavity of patients who underwent surgical repair because of oblique inguinal hernia,

incisional hernia, or appendicitis at the Second Affiliated Hospital of Zhejiang University School of Medicine. All the patients for scWAT collection were checked for full recovery from acute injury and confirmation of the absence of inflammation before surgery. Fat samples from subjects with malignant tumors were excluded. All the tissue samples were freshly collected and immediately frozen in liquid nitrogen. Human blood samples were collected from subjects with differing BMIs, while subjects with malignant tumors, organic lesions, or having previously undergone surgery within several months to half a year were excluded. We are not aware of any potential self-selection bias or other biases during the recruitment of human research participants.

Ethics oversight

The studies on human skeletal muscle and scWAT were approved by the First People's Hospital of Lianyungang City (Approval number: LW-20180321001), and the Second Affiliated Hospital of Zhejiang University School of Medicine (Approval number: 2020-528), respectively. The study on human plasma was approved by the Second Affiliated Hospital of Soochow University (Approval number: JD-LK-2020-038-01). Only the participants who had provided written informed consent were included in the study.

Note that full information on the approval of the study protocol must also be provided in the manuscript.

Field-specific reporting

Please select the one below that is the best fit for your research. If you are not sure, read the appropriate sections before making your selection.

☒ Life sciences ☐ Behavioural & social sciences ☐ Ecological, evolutionary & environmental sciences

For a reference copy of the document with all sections, see nature.com/documents/nr-reporting-summary-flat.pdf

Life sciences study design

All studies must disclose on these points even when the disclosure is negative.

Sample size

Sample sizes were indicated in the legends of Figures and Supplementary Figures. No statistical method was used to pre-determine the sample size. Sample size for animal experiments was determined based on generally expected variations of metabolic parameters and typical sample size for metabolic studies to achieve statistically significant and reproducible results as documented in our previous publications (such as Meng ZX et al. Nature Medicine 2013, PMID: 23563706; Meng ZX et al. Molecular Cell 2017, PMID: 28475869; Meng ZX et al. Diabetes 2018, PMID: 29092888; Kong Q et al. Diabetes 2022, PMID: 35822944; Wang RR et al. J Exp Med 2022, PMID: 35652891), and studies from other laboratories shown in the literature. For animal studies, 4-15 mice per group were used. Sample sizes for cell culture studies were determined based on previous laboratory experience and preliminary experiments. At least 3 biological replicates per group were included to enable statistical analysis. RNA-Seq was performed using total RNA samples from two or three biologically independent animals in each genotype or treatment. The exact sample size number for each group was indicated in the figure legend.

Data exclusions

On rare occasions, mice in bad health conditions, such as severe fighting wounds or exhibiting sickness, according to the predefined criteria listed in the IACUC were excluded.

Replication

All the animal experiments were independently repeated at least twice. The cell culture experiments were independently repeated at least 3 times. All additional replication attempts were successful.

Randomization

Animals were randomly assigned to the experimental and control groups.

Blinding

For experiments including RNA-Seq, CLAMS studies, tissue section preparation for H&E staining, H&E staining data quantification, and mass spectrometry, data acquisition was performed in a blinded fashion as additional staff and support were introduced. For other experiments related to mouse models, investigators were not blinded because the investigators needed to know exactly the genotype or treatment for each mouse when group were allocated, and no blinding can also help to avoid interference or cross-contamination between groups inadvertently during sample preparation; For other experiments related to cell models, investigators were not blinded because the investigators needed to know the cell type for culture and the specific treatment for each group.

All the experiments were performed by strictly following standard laboratory procedures. Proper controls were included in each experiment, and data were collected under the same conditions for both control and experimental groups.

The investigators were not blinded during data analysis because they cannot perform the statistical analysis between treated group(s) and the control group and thus make the final conclusion if they are blinded to each group.

Reporting for specific materials, systems and methods

We require information from authors about some types of materials, experimental systems and methods used in many studies. Here, indicate whether each material, system or method listed is relevant to your study. If you are not sure if a list item applies to your research, read the appropriate section before selecting a response.

Materials & experimental systems

n/a	Involved in the study
☐	☒ Antibodies
☐	☒ Eukaryotic cell lines
☒	☐ Palaeontology and archaeology
☐	☒ Animals and other organisms
☒	☐ Clinical data
☒	☐ Dual use research of concern

Methods

n/a	Involved in the study
☒	☐ ChIP-seq
☒	☐ Flow cytometry
☒	☐ MRI-based neuroimaging

Antibodies

Antibodies used

Rabbit polyclonal antibody to Musclin, generated with mouse Musclin protein and affinity purified (Abcam); total OXPHOS rodent WB antibody cocktail (1:1000, ab110413), and antibodies to Pgc1(α+β) (1:1000, ab72230), from Abcam; antibody to GAPDH (1:1000, sc-25778), from Santa Cruz; antibodies to phospho-PKA substrates (1:1000, 9624), HSL (1:1000, 4107s), phospho-HSL (ser660) (1:1000, 4126s) and Hsp90 (1:1000, C45G5) from Cell Signaling; antibodies to Flag (1:1,000, M2, A8592) and α-tubulin (1:1,000, T6199), from Sigma; antibody to Tfr1 (1:1000, 13-6800), from Invitrogen; antibody to Ucp1 (1:1000, UCP11-A) from Alpha Diagnostic; antibody to Adrb3 (1:1000, A8607), from Abclonal; antibody to His tag (1:1000, A186s), from GenScript; anti-rabbit (1:10000, A6154) or anti-mouse (1:10000, A4416) IgG (whole molecule)-Peroxidase secondary antibodies produced in goat, from Sigma.

Validation

All antibodies except Musclin (rabbit) are commercially available and have been verified by the manufacturers according to the immunoblots and/or images on their websites.
Validation of Musclin has been done in Abcam and our own laboratory using positive control and negative control samples.

anti-Pgc1(α+β) (Abcam, ab72230, 1:1000 for immunoblot)
https://www.abcam.com/pgc1-alpha-beta-antibody-ab72230.html
validated by other users, 34 citations in the company website

Total OXPHOS Rodent WB Antibody Cocktail (Abcam, ab110413, 1:1000 for immunoblot)
https://www.abcam.com/total-oxphos-rodent-wb-antibody-cocktail-ab110413.html
validated by other users, 842 citations in the company website

anti-GAPDH (Santa Cruz Biotechnology, sc-25778, 1:1000 for immunoblot)
https://www.scbt.com/p/gapdh-antibody-fl-335?requestFrom=search
validated by other users, 618 citations in the company website

anti-phospho-PKA substrates (Cell Signaling, 9624, 1:1000 for immunoblot)
https://www.cellsignal.com//products/primary-antibodies/phospho-pka-substrate-rrxs-t-100g7e-rabbit-mab/9624
validated by other users, 192 citations in the company website

anti-HSL (Cell Signaling, 4107s, 1:1000 for immunoblot)
https://www.cellsignal.com/products/primary-antibodies/hsl-antibody/4107?site-search-type=Products&N=4294956287&Ntt=4107s&fromPage=plp&_requestid=245306
validated by other users, 301 citations in the company website

anti-phospho-HSL (ser660) (Cell Signaling, 4126s, 1:1000 for immunoblot)
https://www.cellsignal.com/products/primary-antibodies/phospho-hsl-ser660-antibody/4126
validated by other users, 205 citations in the company website

anti-Hsp90 (Cell Signaling, C45G5, 1:1000 for immunoblot)
https://www.cellsignal.cn/products/primary-antibodies/hsp90-c45g5-rabbit-mab/4877
validated by other users, 393 citations in the company website.

anti-Flag (sigma, M2 A8592, 1:1,000 for immunoblot)
https://www.sigmaaldrich.cn/CN/zh/product/sigma/a8592?context=product
validated by other users, 1046 citations in the company website

anti-α-tubulin (Sigma, T6199, 1:1,000 for immunoblot)
https://www.sigmaaldrich.cn/CN/zh/product/sigma/t6199?context=product
validated by other users, 1655 citations in the company website

anti-Tfr1 (Invitrogen, 13-6800, 1:1,000 for immunoblot)
https://www.thermofisher.cn/cn/zh/antibody/product/Transferrin-Receptor-Antibody-clone-H68-4-Monoclonal/13-6800
validated by other users, 548 citations in the company website

anti-Ucp1 (Alpha Diagnostic, UCP11-A, 1:1000 for immunoblot)
https://www.labome.com/product/Alpha-Diagnostics/UCP11-A.html
validated by other users, 10 citations in the company website.

anti-Adrb3 (Abclonal, A8607, 1:1000 for immunoblot)
https://abclonal.com/catalog-antibodies/ADRB3RabbitpAb/A8607

validated by other users, 3 citations in the company website.

anti-His tag (GenScript, A00186S, 1:1000 for immunoblot)
https://www.genscript.com/antibody/A00186S-THE_His_Tag_Antibody_mAb_Mouse.html
validated by other users, 65 citations in the company website.

anti- rabbit IgG (whole molecule)-Peroxidase secondary antibody (Sigma, A6154, 1:10000 for immunoblot)
https://www.sigmaaldrich.cn/CN/en/product/sigma/a6154
validated by other users, 677 citations in the company website.

anti-mouse IgG (whole molecule)-Peroxidase secondary antibody (Sigma, A4416, 1:10000 for immunoblot)
https://www.sigmaaldrich.cn/CN/en/product/sigma/a4416
validated by other users, 799 citations in the company website

Eukaryotic cell lines

Policy information about cell lines and Sex and Gender in Research

Cell line source(s)	HEK293T, C3H10T1/2, C2C12 and HUVEC cell lines were obtained from ATCC. Expi293F cell line was obtained from ThermoFisher. AAV293 cells were obtained from Agilent. The primary cell lines and cells derived from human participants or vertebrate models were not involved in this study.
Authentication	The authentication of C3H10T1/2 and C2C12 cells were confirmed through cell morphology and global mRNA and protein expression analyses such as RNA-Sequencing, qPCR, and/or western blotting, after fully differentiation. HEK293T, AAV293 and Expi293F cells were authenticated and routinely used in our previous studies (such as Meng ZX et al. Nature Medicine 2013, PMID: 23563706; Kong Q et al. Diabetes 2022, PMID: 35822944; Wang RR et al. J Exp Med 2022, PMID: 35652891; Zhang P et al. Cell Metabolism 2022, PMID: 35973424). HUVEC cell line was provided by Dr. Nan Xu (Henan University), and has been authenticated and successfully used in their previous study (Hu WP et al. Acta Physiol 2021, PMID: 33835704).
Mycoplasma contamination	The cell lines have been tested to be negative for mycoplasma contamination.
Commonly misidentified lines (See ICLAC register)	No commonly misidentified lines were used in this study.

Animals and other research organisms

Policy information about studies involving animals; ARRIVE guidelines recommended for reporting animal research, and Sex and Gender in Research

Laboratory animals	All the C57BL/6J wild-type mice were obtained from GemPharmatech (Nanjing, China). Skeletal muscle-specific Musclin transgenic (MCK-Musclin) mice were generated by pronuclear microinjection with a full-length Musclin coding sequence placed downstream of the 4.8-Kb murine MCK promoter (Sternberg EA et al. Mol Cell Biol. 1988, PMID: 3405222), and were backcrossed to C57BL/6J for at least eight generations to generate stable transgenic lines. Skeletal muscle-specific Musclin knockout (Musclin-MKO) mice were generated by breeding Musclin flox/flox mice with MLC-Cre mice (Bothe GW et al. Genesis 2000, PMID: 10686620). Tfr1 flox/flox mice (JAX stock #028363) and Adipoq-CreERT2 mice (JAX stock #025124), were shared by Dr. Liwei Xie (Guangdong Institute of Microbiology). Adipoq-Cre (JAX stock #028020) mice were generously provided by Dr. Fudi Wang (Zhejiang University). The ob/ob (leptin-deficient) and db/db (leptin receptor-deficient) mice were purchased from the Jackson Laboratory. All mouse strains used in this study were born at the expected Mendelian ratios with normal fertility.<br><br>Mice were housed in 12/12 h light/dark cycles with a humidity of 50-60%, and fed with either a regular chow diet (10 kcal% fat, 70 kcal% carbohydrate, and 20 kcal% protein; 1010088; Jiangsu Xietong Pharmaceutical Bio-engineering Co., Ltd.) or an HFD (60 kcal% fat, 20 kcal% carbohydrate, and 20 kcal% protein; D12492; Research Diets). The temperature and humidity of all animal rooms were electronically monitored and regulated. Mice were bred and housed at 23-24°C except for cold exposure assay. All chow diet-fed mice used in this study were between 2 and 8 months old. For AAV transduction, 8-week-old male C57BL/6J mice were used. For diet-induced obesity in mice, HFD feeding begins at 12 weeks old.
Wild animals	The study did not involve any wild animals.
Reporting on sex	In the metabolic studies involving HFD-induced obese mice, male mice were used since female mice don't gain as much body weight as male mice do based on our experience on the metabolic studies as documented in our previous publications (such as Meng ZX et al. Nature Medicine 2013, PMID: 23563706; Meng ZX et al. Molecular Cell 2017, PMID: 28475869; Meng ZX et al. Diabetes 2018, PMID: 29092888; Kong Q et al. Diabetes 2022, PMID: 35822944; Wang RR et al. J Exp Med 2022, PMID: 35652891), and studies from other laboratories in the metabolism field shown in the literature. For the cold exposure study shown in Fig. 5d and liver metabolic gene expression studies upon AAV transduction in Supplementary Fig. 4d, female mice were also used. And the results were similar to those obtained using male mice.
Field-collected samples	The study did not involve samples collected from the field.
Ethics oversight	All animal studies were performed in compliance with the Guide for the Use and Care of Laboratory Animals by the Medical Experimental Animal Care Committee of Zhejiang University. All animal studies were performed following the protocols approved by

the Animal Ethics Committee of Second Affiliated Hospital, School of Medicine, Zhejiang University (Approval number:2019-90).

Note that full information on the approval of the study protocol must also be provided in the manuscript.
